# Handgrip Strength and Anthropometry in Parkinson's Disease at Diagnosis

**DOI:** 10.1155/2022/1516807

**Published:** 2022-07-02

**Authors:** Lena Håglin, Birgitta Törnkvist, Mona Edström, Sofia Håglin, Lennart Bäckman

**Affiliations:** ^1^Department of Public Health and Clinical Medicine, Family Medicine, Umeå University, SE-901 87, Umeå, Sweden; ^2^Department of Clinical Sciences, Neurosciences, Umeå University, SE-901 87, Umeå, Sweden

## Abstract

**Objectives:**

To investigate how age, malnutrition, and the level of plasma cortisol and phosphate in patients with Parkinson's disease (PD) at time of diagnosis are associated with body composition and handgrip strength in males and females compared to controls. *Materials & Methods*. This cross-sectional study includes baseline data from a cohort of newly diagnosed patients with Parkinson's disease (*N* = 75; *M*/*F* = 41/34) in the New Parkinsonism in Umeå study (NYPUM). Body Impedance (BIS), handgrip strength (HGS) assessments, and evaluation of risk for malnutrition (Mini Nutritional Assessment (MNA) score) and cognitive performance (Mini-Mental State Examination (MMSE)) were performed at time of PD diagnosis.

**Results:**

Low fat-free mass index (FFMI), MNA score, and a high Unified Parkinson's Disease Rating Scale (UPDRS-total and UPDRS-III) were associated with high daytime levels of P-cortisol in total PD population but not in controls. Partial correlations reveal that high fat mass percent (FM (%)) and low FFMI were associated with older age in males with PD but not females with PD. Risk of malnutrition was associated with P-cortisol in males but not in females with PD (*r* = −0.511, *P*=0.001, and *n* = 41 and *r* = −0.055, *P*=0.759, and *n* = 34, respectively). Multiple linear regressions show that an interaction between P-cortisol and P-phosphate, older age, and high UPDRS-III score were associated with HGS in total patient population and males but not females.

**Conclusions:**

Age- and disease-associated risk factors that decrease muscle mass and HGS and increase FM (%) in patients with PD differ between males and females by an association with levels of cortisol and phosphate.

## 1. Introduction

Loss of muscle strength due to muscle wasting may be an early and strong risk factor for debility in PD patients and grip strength could serve as an effective determinant for muscle activity in patients with PD [1]. Aging, environmental stress [2], and malnutrition are associated with low handgrip strength [3]. At high age, patients with PD have lower body weight and amount of body fat than healthy controls with nonsignificant difference in the intake of energy and macronutrients [4].

To increase understanding of mechanisms behind changes in anthropometry and functional decline, it is important to highlight the influence from stress and cortisol levels [5] and to consider the difference between males and females [2]. Glucocorticoids have been proposed to be involved in the aetiology of PD by facilitating neuronal degeneration and influence progression of the disease [5].

It is challenging to assess whether qualitative changes in muscle mass, amount of body fat, and muscle metabolism observed in PD [6, 7] are associated with specific pathological mechanisms or are a part of normal aging [8]. Previous studies have shown differences in both disease progress and anthropometry between males and females with PD. Hence, it is important to signify stratification for sex in studies of multifactorial aspects on aetiology and progress of PD.

Here, we investigate how age, risk of malnutrition, and the level of P-cortisol and P-phosphate in patients at time of diagnosis are associated with body composition and handgrip strength in males and females at time of PD diagnosis compared to controls.

## 2. Materials and Methods

### 2.1. Total Population

All suspected cases of idiopathic Parkinsonism in Västerbotten county were referred to the neurological department at Umeå University Hospital (Northern Sweden). From January 2004 through April 2009, 186 Parkinsonism cases were identified [9]. Clinical criteria for PD diagnosis were evaluated by a specialist in movement disorders (blinded to the assessment of the previous examiner) from a videotape of the patients undergoing the Unified Parkinson's Disease Rating Scale (UPDRS-III scale) examination. A patient was included if both examiners judged that the patient had fulfilled the clinical criteria for Parkinson´s disease (*N* = 151) according to the UK Parkinson's Disease Society Brain Bank (UK PDSBB) criteria [10].

### 2.2. Study Population

This cross-sectional study includes baseline data from a cohort of patients with Parkinson's disease diagnosed in the New Parkinsonism in Umeå study (NYPUM). The data collected in the PD patient population (*N* = 75; *M*/*F* = 41/34) at diagnosis were compared with data from a control group (*N* = 24; *M*/*F* = 12/12) selected by announcement, according to the age and gender of the first 50 PD patients diagnosed in the NYPUM study.

At time of diagnosis, a few patients were in a clinical condition in need for deep brain stimulation (DBS). These patients were, after diagnosis with worse progress, retrospectively excluded (*N* = 6). Another reason for study dropouts was lack of time or if a patient was enrolled in other studies on immobility and mortality.

Of this final patient population (*N* = 137), 55% took part in the nutritional status assessment at baseline (*n* = 75; *M*/*F* 41/34).

### 2.3. Disease Severity and Medication

Disease severity (i.e., the motor symptom severity) was assessed using the Unified Parkinson's Disease Rating Scale (UPDRS-total and UPDRS–III score), [11] and the Hoehn and Yahr (H&Y) staging scale [12]. The UPDRS scoring was performed when the patients were in the ON phase of the disease and repeated when they started dopaminergic treatment. Clinical evaluation of motor function of upper extremities was performed, and symptoms were assigned unilaterally on either left or right side or bilaterally (symmetric).

The levodopa equivalent daily dose (LEDD) was calculated using a conversion factor for each of the anti-Parkinson medications [13]. Two patients had started anti-Parkinson treatment at time of diagnosis, 13 patients had started anti-Parkinson medication after diagnosis but before blood sampling, and 60 patients started anti-Parkinson medication after blood sampling.

Cognitive ability was evaluated using the MMSE score, a screening instrument assessing cognitive ability, including memory, orientation in time, and place, ability to follow simple commands, and language [14].

### 2.4. Anthropometry and Handgrip Strength

At time of diagnosis, body weight (kg) using a digital scale and height (cm) using a stadiometer were measured and body mass index (BMI) was calculated. Muscle strength measurements of left and right hands (handgrip strength (HGS)) were performed using a JAMAR dynamometer [15], and the patients were instructed to squeeze the device once for 3–5 seconds. This study uses a cut-off for handgrip strength independent of BMI (≤25.8 kgf for males and ≤17.4 kgf for females) [8].

### 2.5. Bioelectrical Impedance Spectroscopy

Assessments of body composition compartments were performed using a multifrequency Bioelectrical Impedance Spectrum (BIS) analyser and Cole-Cole modelling software (hydra extracellular water/intracellular water (ECW/ICW), Hydra model 4200, Xitron Technologies, San Diego, CA). The body weight (kg), height (cm), and sex (male or female) were entered into the BIS program and two pairs of electrodes were applied 5 cm apart, one pair on the right hand/wrist and one pair on the right foot/ankle. Cables with clips were connected to the electrodes.

Measurements of fat-free mass (FFM, (kg)), ECW (l), and ICW (l) were registered. The results were used to discriminate between ECW and ICW in muscle mass and to calculate ECW/ICW ratio and total body water (TBW = ECW + ICW). Normalized FFM by height (FFMI; FFM (kg/height), (m^2^)) and ICW were used as proxies for muscle mass [16]. Fat mass, the difference between body weight and FFM, was calculated in percent (FM (%)). The cut-offs used for FFMI to assess low amount of muscle mass were ≤17.4 for males and ≤15.0 for females, based on a patient population [17].

### 2.6. Nutritional Status and Physical Activity

The Mini Nutritional Assessment (MNA) score, an international validated screening tool, was used to assess risk of malnutrition [18]. The MNA screening consists of 18 questions regarding health, anthropometry, and diet. An MNA total score between 24 and 30 points indicates optimal nutritional status, a total score between 17 and 23.5 points indicates a risk for malnutrition, and a total score less than 17 points indicates malnutrition. Initial questions refer to MNA-initial (MNA-SF; short form) with a score from 7 to 14 points and was included in addition to final score as a complement to MNA-total. The physical activity level (PAL) was registered by the patient and calculated for different activities, which has been described in detail in a previous publication [19].

### 2.7. Biochemical Analysis

The blood was collected between 8 am and 4 pm in a nonfasting condition, when the patients were admitted for assessment of nutritional status and neuropsychological tests.

The plasma samples were used to measure cortisol (P-cortisol, (nmol/l)), phosphate (P-phosphate, (mmol/l)), and albumin (P-albumin, (g/l)) levels. Thus, cortisol levels correspond to daytime levels and not early morning or evening levels. Therefore, a circadian rhythm for cortisol could not be assessed. Analysis was performed using clinical routines in an accredited laboratory at Umeå University Hospital. Plasma cortisol levels were analysed using Elecsys Cortisol reagent on a cobas e 601 analyser. P-phosphate and P-albumin were analysed using Vitros PHOS Slides, Vitros ALB Slides, and Vitros TRFRN reagent on an Ortho Vitros 5.1 FS analyser.

### 2.8. Statistics

An independent two-tailed test was used to compare characteristics between either patients and controls or male and female patients. Bivariate and partial correlations were analysed using Pearson's correlation coefficient, [20] with adjustment for UPDRS-III, BMI, PAL, and MNA-total in the partial correlation. Separate regression models were conducted for each outcome. Multiple linear regression models with ECW/ICW ratio, TBW, FFMI, FM (%), and left and right HGS as dependent variables were used to study association with P-cortisol and P-phosphate and an interaction between P-cortisol and P-phosphate. Adjustment for age, sex, and UPDRS-III was done in the regression model. We tested associations between normalized and unadjusted raw HGS with asymmetry from upper body disturbances (left side, right side, symmetric) by one-way ANOVA. The program Predictive Analytics Software (PASW Statistics, version 18.0.3, SPSS Inc., Chicago IL, USA) was used for the analysis, and imputations for missing variables were done by expectation maximization algorithm using missing value analysis. We did imputations on four variables with one missing each, on one variable with two missing each, and on three variables with three missing each. No missing variables were found in 90% of males and in 76% of females.

## 3. Results

Clinical characteristics and anthropometric indices of the study population comprising male and female patients with PD and controls are presented in Table 1. The prevalence of low handgrip strength in PD patients was 27% in males (11/41) and 42% in females (14/34), compared with 7% in males (1/12) and 38% in females (5/12) among controls. Low FFMI was observed in both males and females with PD, 80% (33/41) and 72% (25/34), respectively. In controls, low FFMI could be detected in 67% (8/12) of males and in 79% (9/12) of females.

The distribution of initial manifestations of clinical symptoms in the upper extremities, with three symptomatic distribution patterns, comparing left side, right side, and bilateral engagement, did not differ between males and females (*P*=0.680) with PD. Mean values for left and right HGS did not differ between the distribution patterns (*P*=0.339). However, UPDRS-total score was associated with distribution of clinical symptoms (*P*=0.009), where higher scores were associated with bilateral symptoms (41.1 ± 11.7) and lower scores with isolated left sided disturbances (29.9 ± 10.8). Next, we studied the influence of the level of P-cortisol and P-phosphate on HGS and body composition. The mean levels of P-cortisol were not different between patients who started anti-Parkinson medication before compared to after blood sampling (412 ± 165 nmol/l (*n* = 13) and 385 ± 139 nmol/l (*n* = 60), respectively (*P*=0.537)). A higher risk for malnutrition (lower MNA score) was shown in the PD patients as compared with controls (Table 1).

Bivariate correlations show that MNA-total, MNA-initial and MNA-final scores, FFMI, ICW, and P-phosphate were negatively associated with P-cortisol in males but not in females among patients with PD (Table 2). In males with PD, UPDRS-III, FM (%), and ECW/ICW ratio were positively associated with P-cortisol, in contrast to lack of association in females with PD.

Bivariate correlation analyses within the groups of patients and controls revealed that UPDRS-total and UPDRS-III, P-phosphate, ICW, TBW, and FFMI correlated with P-cortisol in PD (Table 2). None of the studied variables correlated with P-cortisol in controls. P-albumin and P-cortisol were not associated in any of the groups.

Partial correlations were performed with adjustments for sex, BMI, PAL, and MNA-total score as well as UPDRS score for patients with PD. ECW/ICW ratio, TBW, and bilateral HGS correlated with age in the population of PD patients. In males with PD, FFMI, TBW, and bilateral HGS correlated negatively, while ECW/ICW ratio and FM (%) correlated positively with age (Table 3). In controls, right HGS and TBW correlated negatively with age. Multiple linear regressions were performed with left and right HGS as the dependent variable and an interaction between P-cortisol and P-phosphate was included in the regression models if significant. High levels of P-cortisol and high levels of P-phosphate were associated with high HGS in patients with PD. An interaction between P-cortisol and P-phosphate was negatively associated with left and right HGS in total patient population. Age was negatively associated with bilateral HGS in patients and controls and positively associated with ECW/ICW ratio and FM (%) in patients but not in controls. Multiple linear regression analyses revealed a negative association between the level of P-cortisol and both TBW and FFMI in patients. UPDRS-III was positively associated with ECW/ICW ratio but negatively with TBW and FFMI in patients. BMI and sex were similarly associated with TBW, FFMI, and FM (%) in patients and controls.

The interaction between P-cortisol and P-phosphate was not associated with ECW/ICW ratio, TBW, FFMI, or FM (%) neither in cases nor in controls and therefore was not included in the analysis. The level of P-cortisol was negatively associated with TBW and FFMI in patients with PD but not in controls. UPDRS-III was positively associated with ECW/ICW ratio but negatively with TBW and FFMI in PD.

## 4. Discussion

In this study, we have shown sex specific associations between anthropometric variables and the level of P-cortisol at time of diagnosis of PD. Higher UPDRS scores and age were associated with low FFMI and high FM (%) in males but not in females. We used BIS to discriminate between ECW and ICW from total body measurements, which reveals that both age and UPDRS-III correlated with ECW/ICW ratio, indicating that aging and disease progress have common denominators for lower ICW and/or higher ECW distribution.

Muscle strength and gait speed have been shown to be associated with ECW/ICW ratio, [1, 7, 21] together with the age-related disturbance in this study (Table 3) which indicates that aging and PD contribute to these anthropometric disturbances. Furthermore, these parameters are also associated with lower HGS. In this study, we have observed that age correlates with ECW/ICW ratio in the total PD population but not in controls, thus indicating disease-specific associations with age. Moreover, analysis of sex differences revealed that low FFMI and high FM (%) were age related in males but not in females with PD.

Patients with more extensive motor disturbance indicated by higher UPDRS-III had lower amount of FFM at diagnosis, suggesting an effect from reduced muscle mass on motor function by the aging process and/or by malnutrition indicated by lower MNA in patients. In the present study, association between FFMI and P-cortisol could be observed in patients but not in controls, indicating a pathological mechanism with increased stress which may contribute to reduced muscle mass in patients with PD.

Recently, it was discovered that multifactorial aspects of PD aetiology including aging, malnutrition, and stress were the strongest risk factors related to prodromal mechanisms associated with a disturbed hypothalamic-pituitary-adrenal (HPA) axis and high levels of P-cortisol [5]. However, whether this mechanism is correlated to the previously described increased risk for sarcopenia with age and malnutrition in patients with PD needs to be determined. In this study, we show that age-associated lower HGS was seen in both males and females, whereas a lower FFMI and higher FM (%) with age were observed only in males. More research is needed to differentiate between disease-related pathological anthropometric disturbances and normal aging and further investigate potential sex-specific mechanisms. High activity in the hypothalamic-pituitary-adrenal axis (HPA axis, e.g., increase in cortisol levels) may originate from both stress-related lifestyle habits, malnutrition/low energy intake, and increased age [2]. An inverse association between MNA and levels of cortisol in patients but not in controls suggests that malnutrition is involved in a “stress-related” condition in PD. This hypothesis is supported by the higher risk for malnutrition in patients compared to controls in the present study. Daily stress has been indicated to exacerbate the progression of Parkinson's disease, a result that may originate from endocrine dysregulation of the HPA axis [22]. Moreover, increased acrophase, amplitude, and area under the curve for cortisol are associated with gait disturbance in patients with PD [23]. A life-long connection between muscle strength and PD is indicated by results from a study in males which showed low muscle strength three decades before the clinical onset of PD [24]. Risk factors for neurogenic sarcopenia in PD may be present years before diagnosis and associated with lost motor neurons [25], faster progress with longer disease duration, and more motor impairment [4, 6].

The main results in this study reveal that increased HGS was associated with a higher level of either P-cortisol or P-phosphate in patients with PD but not in controls. The negative association with the interaction between P-cortisol and P-phosphate indicates that the level of P-cortisol affects the influence of P-phosphate on muscle strength. As P-phosphate differs between males and females [26] and is known to correlate inversely with P-cortisol, [27], we tested for associations between variables for body composition and P-cortisol and P-phosphate interaction to see if muscle strength and body composition were affected in patients with PD. We could see that high level of P-phosphate may improve HGS when P-cortisol is low. Inversely, this interaction also suggests that high P-cortisol improves HGS even with a low P-phosphate level. At a phosphate level of around 1.0 mmol/L and high P-cortisol, HGS increases; conversely, it decreases at low P-cortisol levels.

It has been shown that muscle weakness may be a result of low levels of phosphate [28] and that increased intestinal absorption of phosphate improves muscle and liver metabolism, diaphragmatic endurance, and recovery from fatigue [29]. Here we report a sex-specific disease-related finding where males with PD have increased level of P-cortisol in relation to lower level of P-phosphate with increased age. These results may explain a part of a sarcopenic risk condition indicated by low handgrip strength. In contrast, P-cortisol was negatively associated with low HGS. This suggested that association is further supported by studies showing that neuromuscular disturbances may origin from hypophosphatemia [30] and that restoring a low level of phosphate can improve muscle strength [31].

Clinical studies reveal that neurodegeneration with loss of motor units predisposes muscle wasting in PD and is present prior to signs of sarcopenia [25, 32]. Sarcopenia during early stages of PD is linked to motoric deficits observed already during the prodromal phase of the disease [25].

Taken together, this can be viewed as an indicator of an association between age, UPDRS-III, and HGS which supports the existence of an “extended neurodegenerative overlap syndrome” [25]. We hypothesize that an interaction between P-cortisol and P-phosphate, at time of diagnosis, reflects acquired stress observed by high P-cortisol, malnutrition, and high age, which could contribute to loss of muscle strength long before diagnosis. Elucidation of metabolic interactions as indicators for increased risk for onset of PD can identify additional mechanisms behind loss of muscle strength during the prodromal period.

The results from the present study reveal that P-cortisol and P-phosphate are important biomarkers for detecting dysfunctions in the strength of muscle revealed by HGS. Furthermore, we show the need to differentiate between anthropometrical measures and muscle strength when assessing risk factors for the pathological age-related deranged body composition and loss of muscle strength in the early stage of PD. In patients with PD, an increase in P-cortisol, low P-phosphate, and excess amount of body fat, in addition to low HGS, can be used as indicators for reduced muscle strength and to assess early clinical risks for disability and the disease progress.

The NYPUM project has several strengths. It collects data for improved clinical assessments and optimal diagnoses, which included a yearly follow-up to secure the setting of a PD diagnosis. In addition, all nutritional data were collected by one professional dietician. The weakness of the NYPUM project is the rather small population, which limits the statistical power, and lack of a variable for lifetime stress limits discussion on origin of a disturbed HPA axis and high cortisol levels (see Tables 4 and 5)

## 5. Conclusions

We suggest that the mechanisms leading to greater loss of muscle mass expressed as an increase in ECW/ICW ratio and FM (%) but a decrease in FFMI with age in males compared with females may be associated with higher P-cortisol and/or lower P-phosphate levels. An interaction between cortisol and phosphate may have a central role in the understanding of mechanisms related to the aging process with respect to muscle metabolism and therefore loss of muscle strength in patients with PD, stronger in males than in females.

## Figures and Tables

**Table 1:** Clinical characteristics and anthropometric indices of the study population, patients with PD, and controls and for male and female patients at time of PD diagnosis.

	Patients *N* = 75	Controls *N* = 24	*P* value	Male patients *N* = 41	Female patients *N* = 34	*P* value
Age, years	68.9 (8.6)	68.2 (6.6)	0.668	69.8 (8.9)	67.8 (8.2)	0.319
UPDRS-total	34.6 (12.7)	—	—	**37.5 (12.0)**	**30.4 (12.5)**	**0.015**
UPDRS-III	24.9 (10.6)	—	—	**27.4 (9.7)**	**21.9 (10.9)**	**0.023**
H &Y (median)	2.0	—	—	2.0	2.0	0.753
MMSE score	28.7 (1.4)	29.1 (0.8)	0.131	28.5 (1.5)	29.0 (1.2)	0.173
P-cortisol, (nmol/l)	386 (143)	335 (115)	0.113	396 (134)	374 (154)	0.514
P-phosphate, (mmol/l)	1.20 (0.16)	1.23 (0.14)	0.441	**1.15 (0.16)**	**1.26 (0.14)**	**<0.001**
MNA-total	**25.5 (2.4)**	**27.2 (1.5)**	**<0.001**	25.8 (2.4)	25.2 (2.3)	0.272
MNA-initial	12.5 (1.7)	13.0 (1.3)	0.140	12.8 (1.5)	12.2 (1.8)	0.151
MNA-final	**13.1 (1.6)**	**14.2 (1.0)**	**<0.001**	13.0 (1.5)	13.1 (1.7)	0.791
PAL	1.51 (0.19)	1.52 (0.19)	0.947	1.50 (0.17)	1.53 (0.17)	0.425
HGS, left (kgf)	26.5 (10.3)	27.3 (10.5)	0.731	**33.1 (7.9)**	**18.6 (6.3)**	**<0.001**
HGS, right (kgf)	28.1 (10.4)	29.3 (9.7)	0.613	**35.2 (7.5)**	**20.2 (7.2)**	**<0.001**
BMI (kg/m^2^)	26.1 (4.1)	25.6 (4.7)	0.541	26.4 (3.4)	25.9 (4.8)	0.584
ECW (lit)	16.2 (2.7)	16.2 (3.7)	0.950	**17.9 (1.9)**	**14.0 (1.7)**	**<0.001**
ICW (lit)	17.5 (4.8)	17.7 (5.4)	0.815	**19.2 (3.6)**	**15.3 (5.3)**	**<0.001**
ECW/ICW ratio	0.96 (0.16)	0.94 (0.12)	0.534	0.95 (0.15)	0.98 (0.13)	0.386
TBW (lit)	33.6 (6.8)	33.9 (8.9)	0.852	**37.1 (4.9)**	**28.5 (4.4)**	**<0.001**
FFMI (kg/m^2^)	15.4 (2.4)	15.2 (2.8)	0.666	**16.2 (1.7)**	**14.1 (2.2)**	**<0.001**
FM (%)	41.1 (6.0)	40.4 (7.0)	0.641	**38.4 (5.2)**	**45.2 (5.1)**	**<0.001**

UPDRS = Unified Parkinson's Disease Rating Scale. H&Y = Hoehn & Yahr. MNA = Mini Nutritional Assessment. PAL = physical activity level. HGS = handgrip strength (kgf). BMI = body mass index (kg/m^2^). ECW = extracellular water, l. ICW = intracellular water, l. TBW = total body water (ECW + ICW). FFMI = fat-free mass index (kg/m^2^). FM (%) = fat mass percent. The significance level is 95% CI ^∗^*p*<0.05.

**Table 2:** Bivariate correlations *r* (*P* value) between daytime P-cortisol levels and characteristics, including plasma phosphate and body composition variables at time of diagnosis.

	Patients *N* = 75	Controls *N* = 25	Male patients *N* = 41	Female patients *N* = 34
Age, years	0.226 (0.051)	−0.047 (0.826)	**0.393 (0.011)**	0.024 (0.893)
Sex, *M* = 0, *F* = 1	−0.077 (0.514)	0.131 (0.542)	—	—
UPDRS-total	**0.255 (0.028)**	—	0.285 (0.071)	0.202 (0.251)
UPDRS-III	**0.312 (0.006)**	—	**0.332 (0.034)**	0.276 (0.114)
H&Y	0.136 (0.244)	—	0.162 (0.312)	0.120 (0.500)
MMSE	−0.058 (0.618)	0.152 (0.490)	−0.077 (0.633)	−0.010 (0.954)
P-phosphate, (mmol/L)	**−0.355 0.002)**	−0.153 (0.475)	**−0.408 (0.008)**	−0.292 (0.094)
MNA-total	**−0.281 (0.015)**	−0.119 (0.618)	**−0.511 (0.001)**	−0.055 (0.759)
MNA-initial	**−0.308 (0.007)**	−0.029 (0.895)	**−0.332 (0.034)**	−0.320 (0.065)
MNA-final	−0.106 (0.374)	−0.198 (0.403)	**−0.453 (0.003)**	0.331 (0.064)
PAL	−0.209 (0.072)	0.064 (0.771)	**−0.148 (0.357)**	−0.271 (0.121)
HGS, left (kgf)	−0.003 (0.977)	−0.314 (0.145)	−0.089 (0.581)	−0.077 (0.667)
HGS, right (kgf)	−0.033 (0.779)	−0.218 (0.318)	−0.133 (0.407)	−0.117 (0.509)

*Anthropometry*				
BMI (kg/m^2^)	−0.132 (0.260)	−0.082 (0.702)	0.023 (0.886)	−0.259 (0.140)
ECW (lit)	−0.175 (0.132)	−0.187 (0.383)	−0.299 (0.057)	**−0.412 (0.015)**
ICW (lit)	**−0.262 (0.023)**	−0.059 (0.783)	**−0.458 (0.003)**	−0.287 (0.100)
ECW/ICW ratio	0.194 (0.096)	−0.220 (0.302)	**0.362 (0.020)**	0.008 (0.965)
TBW (lit)	**−0.240 (0.038)**	−0.117 (0.585)	**−0.442 (0.004)**	**−0.354 (0.040)**
FFMI (kg/m^2^)	**−0.230 (0.047)**	−0.142 (0.509)	**−0.346 (0.027)**	−0.273 (0.118)
FM (%)	0.153 (0.191)	0.132 (0.537)	**0.513 (0.001)**	0.052 (0.770)

UPDRS = Unified Parkinson's Disease Rating Scale. H&Y = Hoehn & Yahr. MNA = Mini Nutritional Assessment. PAL = physical activity level. HGS = handgrip strength (kgf). BMI = body mass index (kg/m^2^). ECW = extracellular water. ICW = intracellular water. TBW = total body water (ECW + ICW). FFMI = fat-free mass index (kg/m^2^). FM (%) = fat mass percent. The significance level is 95% CI ^∗^*p*<0.05.

**Table 3:** Partial correlations, *r* value (*P* value), between age handgrip strength and anthropometrical indices in all patients and controls and for male and female patients separately. Adjustments were done for sex, BMI, PAL, and MNA-total score in controls and adding UPDR-III in patients.

Variables	Patients *N* = 75	Controls *N* = 24	Male patients *N* = 41	Female patients *N* = 34
HGS, left (kgf)	**−0.397 (0.004)**	−0.402 (0.088)	**−0.352 (0.033)**	**−0.380 (0.038)**
HGS, right (kgf)	**−0.398 (0.004)**	**−0.492 (0.032)**	**−0.369 (0.025)**	−0.290 (0.120)
ECW/ICW ratio	**0.408 (0.003)**	0.336 (0.160)	**0.619 (<0.001)**	**0.536 (0.002)**
TBW	**−0.349 (0.013)**	**−0.515 (0.024)**	**−0.645 (<0.001)**	**−0.505 (0.004)**
FFMI (kg/m^2^)	−0.098 (0.502)	−0.039 (0.873)	**−0.482 (0.003)**	−0.252 (0.178)
FM (%)	0.210 (0.152)	0.052 (0.834)	**0.487 (0.002)**	0.181 (0.338)

HGS = handgrip strength. ECW = extracellular water. ICW = intracellular water. TBW = total body water (ECW + ICW). FFMI = fat-free mass index (kg/m^2^). FM (%) = fat mass percent. The significance level is 95% CI ^∗^*p*<0.05.

**Table 4:** Multiple linear regression models, *B*-value, and *P* value for left and right handgrip strength. Interaction between cortisol and phosphate is included.

	Patients *N* = 75		Controls *N* = 24	
	Handgrip strength			
	Left	Right	Left	Right
BMI (kg/m^2^)	−0.020 (0.916)	0.166 (0.400)	0.203 (0.485)	0.307 (0.260)
Age, years	**−0.332 (<0.001)**	**−0.375 (<0.001)**	**−0.586 (0.007)**	**−0.460 (0.018)**
Sex, *M* = 0, *F* = 1	**−15.470 (<0.001)**	**−15.674 (<0.001)**	**−11.850 (<0.001)**	**−9.464 (<0.001)**
P-cortisol (*μ*mol/l)	**0.125 (0.004)**	**0.113 (0.014)**	−0.021 (0.054)	−0.015 (0.137)
P-phosphate (mmol/l)	**33.418 (0.015)**	**33.504 (0.028)**	0.324 (0.974)	−14.518 (0.130)
UPDRS-III	**−0.185 (0.017)**	−0.094 (0.238)	—	—
Cortisol x phosphate	**−0.102 (0.005)**	**−0.094 (0.013)**	—	—
*R* ^2^	0.680	0.667	0.801	0.801

BMI = body mass index (kg/m^2^). UPDRS = Unified Parkinson's Disease Rating Scale.

**Table 5:** Multiple linear regression models, *B*-value, and *P* value in patients (*N* = 75) and controls (*N* = 24) for ECW/ICW ratio, TBW (ECW + ICW, l), FFMI, and FM (%). Interaction between cortisol and phosphate is excluded (not significant).

	ECW/ICW ratio		TBW		FFMI		FM (%)	
	Cases *N* = 75	Controls *N* = 24	Cases *N* = 75	Controls *N* = 24	Cases *N* = 75	Controls *N* = 24	Cases *N* = 75	Controls *N* = 24
Age, years	**0.007 (<0.001)**	0.007 (0.054)	**−0.141 (0.033)**	**−0.382 (<0.001)**	−0.006 (0.823)	−0.036 (0.303)	**0.135 (0.041)**	0.146 (0.317)
BMI (kg/m^2^)	−0.004 (0.327)	−0.002 (0.577)	**0.515 (<0.001)**	**0.880 (<0.001)**	**0.324 (<0.001)**	**0.413 (<0.001)**	**0.615 (<0.001)**	**0.705 (0.002)**
Sex, *M* = 0 *F* = 1	0.055 (0.121)	0.022 (0.627)	**−9.175 (<0.001)**	**−9.515 (<0.001)**	**−1.961 (<0.001)**	**−1.621 (0.003)**	**7.961 (<0.001)**	**6.169 (0.006)**
P-cortisol (*μ*mol/l)	0.000 (0.662)	0.000 (0.239)	**−0.010 (0.019)**	0.000 (0.978)	**−0.003 (0.040)**	−0.001 (0.675)	0.007 (0.090)	0.006 (0.479)
P-phosphate (mmol/l)	−0.052 (0.652)	−0.124 (0.469)	1.159 (0.759)	6.051 (0.162)	−0.522 (0.727)	0.346 (0.847)	−1.362 (0.713)	−1.852 (0.806)
UPDRS-III	**0.005 (0.006)**	—	**−0.154 (0.007)**	—	**−0.047 (0.034)**	—	0.052 (0.348)	—
*R* ^2^	0.379	0.318	0.641	0.942	0.558	0.887	0.562	0.613

UPDRS = Unified Parkinson's Disease Rating Scale. BMI = body mass index (kg/m^2^). ECW = Extracellular Water, l. ICW = intracellular water, l. TBW = total body water (ECW + ICW). FFMI = fat-free mass index (kg/m^2^). FM (%) = fat mass percent. The significance level is 95% CI ^∗^*p*<0.05.

## Data Availability

Data cannot be shared publicly because of ethical and legal reasons since public availability would compromise patient confidentiality. Data are available upon request if approval from the Regional Ethics Committee is given. Requests may be sent to the corresponding author or to the Regional Ethics Committee, Umeå. The address for such a request is as follows: Regionala Etikprövningsnämnden i Umeå, Samverkanshuset, C/O Umeå Universitet, 901 87 UMEÅ, Sweden (e-mail: epn@adm.umu.se).
